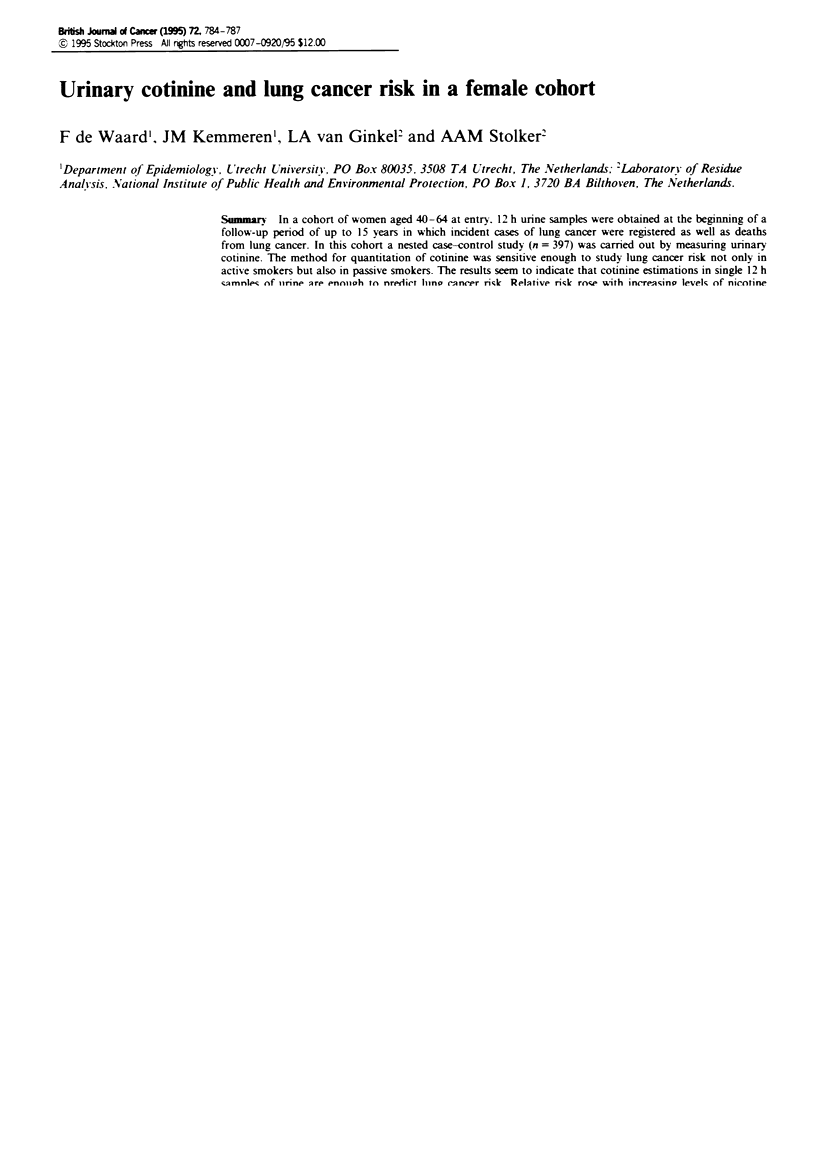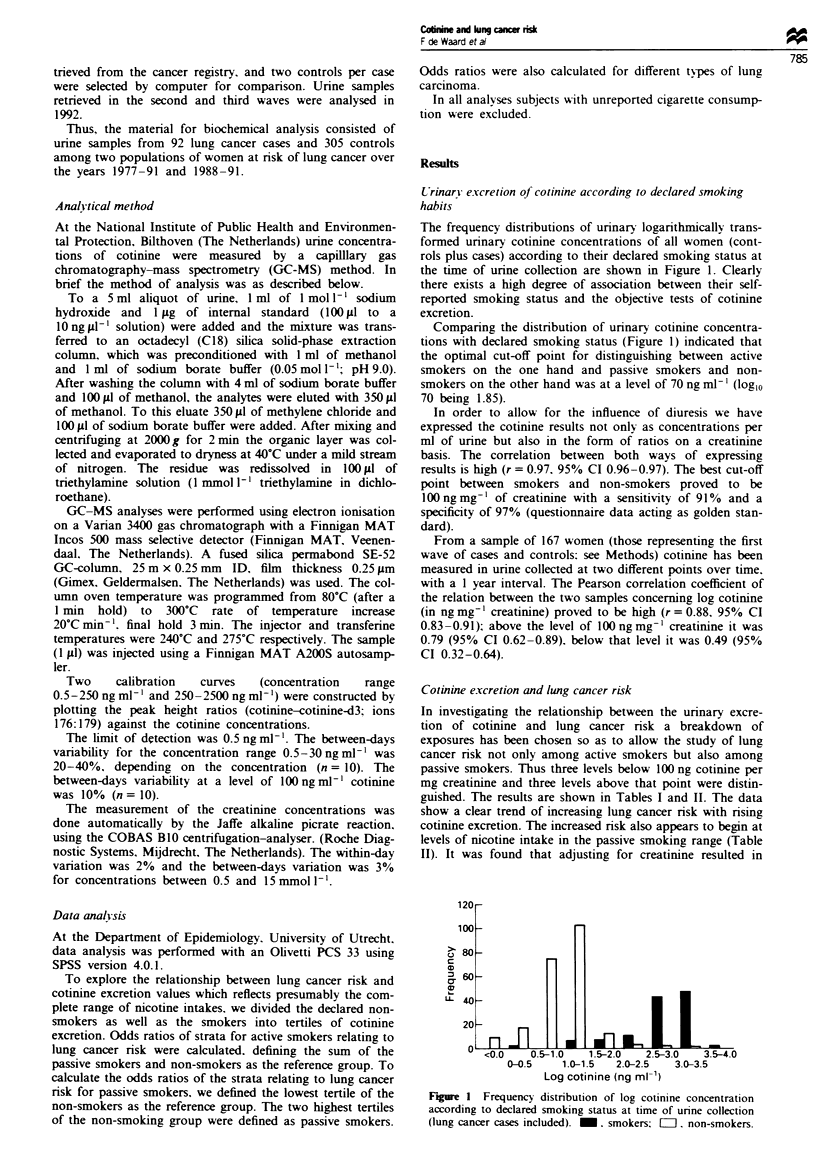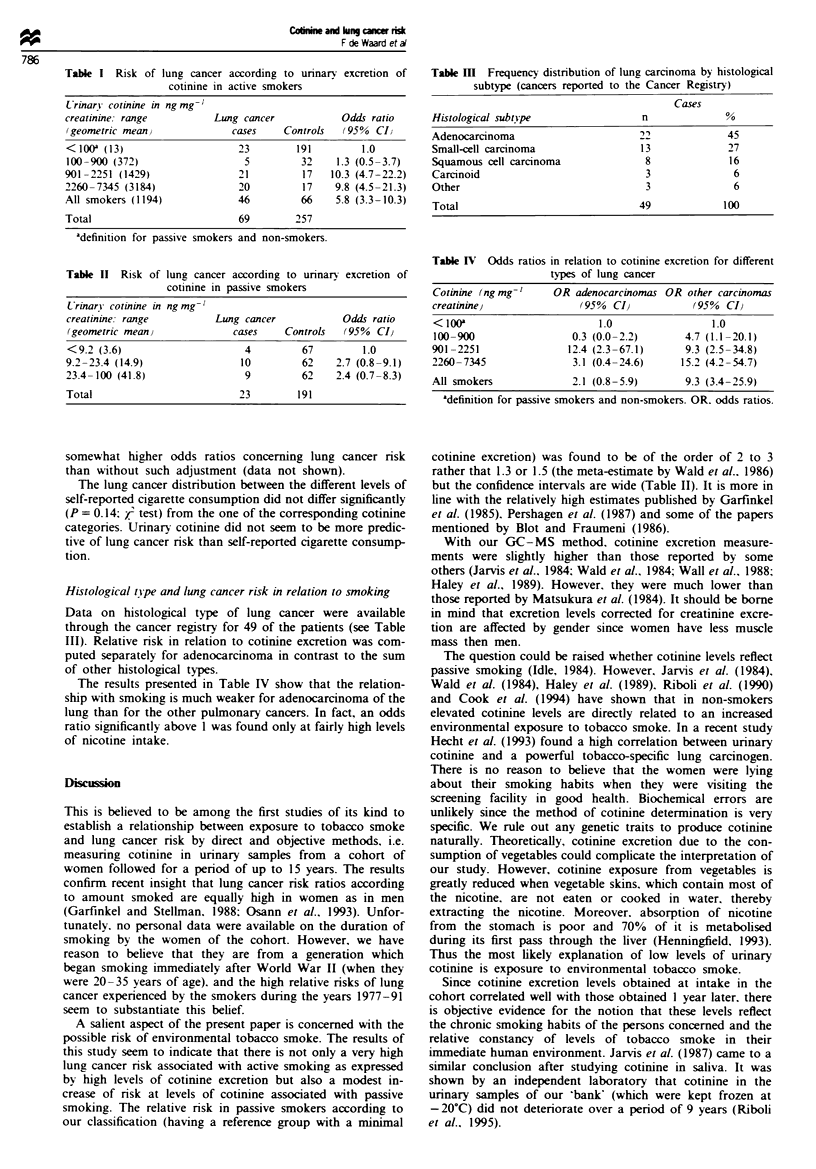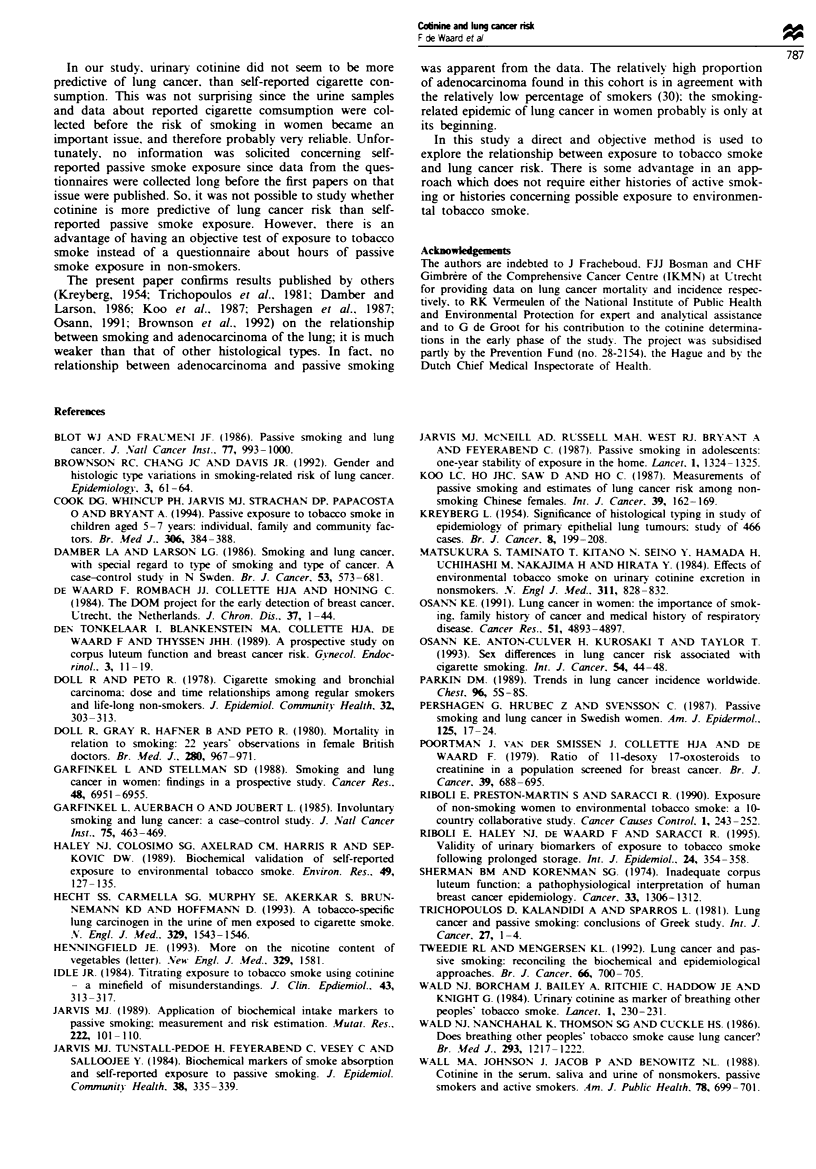# Urinary cotinine and lung cancer risk in a female cohort.

**DOI:** 10.1038/bjc.1995.411

**Published:** 1995-09

**Authors:** F. de Waard, J. M. Kemmeren, L. A. van Ginkel, A. A. Stolker

**Affiliations:** Department of Epidemiology, Utrecht University, The Netherlands.

## Abstract

In a cohort of women aged 40-64 at entry, 12 h urine samples were obtained at the beginning of a follow-up period of up to 15 years in which incident cases of lung cancer were registered as well as deaths from lung cancer. In this cohort a nested case-control study (n = 397) was carried out by measuring urinary cotinine. The method for quantitation of cotinine was sensitive enough to study lung cancer risk not only in active smokers but also in passive smokers. The results seem to indicate that cotinine estimations in single 12 h samples of urine are enough to predict lung cancer risk. Relative risk rose with increasing levels of nicotine intake already in the range associated with passive smoking. The smoking-related risk of adenocarcinoma was much less than that of other lung carcinomas.


British Journal of Cancer (1995) 72, 784-787
© 1995 Stockton Press All rights reserved 0007-0920/95 $12.00

### Urinary cotinine and lung cancer risk in a female cohort

F de Waard[1], JM Kemmeren[1], LA van Ginkel[2] and AAM Stolker[2]

[1]*Department of Epidemiology, Utrecht University, PO Box 80035, 3508 TA Utrecht, The Netherlands;* [2]*Laboratory of Residue Analysis, National Institute of Public Health and Environmental Protection, PO Box 1, 3720 BA Bilthoven, The Netherlands.*

**Summary** In a cohort of women aged 40-64 at entry, 12 h urine samples were obtained at the beginning of a follow-up period of up to 15 years in which incident cases of lung cancer were registered as well as deaths from lung cancer. In this cohort a nested case-control study ($n$ = 397) was carried out by measuring urinary cotinine. The method for quantitation of cotinine was sensitive enough to study lung cancer risk not only in active smokers but also in passive smokers. The results seem to indicate that cotinine estimations in single 12 h samples of urine are enough to predict lung cancer risk. Relative risk rose with increasing levels of nicotine trieved from the cancer registry, and two controls per case were selected by computer for comparison. Urine samples retrieved in the second and third waves were analysed in 1992.

Thus, the material for biochemical analysis consisted of urine samples from 92 lung cancer cases and 305 controls among two populations of women at risk of lung cancer over the years 1977-91 and 1988-91.

### Analytical method

At the National Institute of Public Health and Environmental Protection, Bilthoven (The Netherlands) urine concentrations of cotinine were measured by a capilllary gas chromatography-mass spectrometry (GC-MS) method. In brief the method of analysis was as described below.

To a 5 ml aliquot of urine, 1 ml of 1 mol $l^{-1}$ sodium hydroxide and 1 μg of internal standard (100 μl to a 10 ng $\mu l^{-1}$ solution) were added and the mixture was transferred to an octadecyl (C18) silica solid-phase extraction column, which was preconditioned with 1 ml of methanol and 1 ml of sodium borate buffer (0.05 mol $l^{-1}$; pH 9.0). After washing the column with 4 ml of sodium borate buffer and 100 μl of methanol, the analytes were eluted with 350 μl of methanol. To this eluate 350 μl of methylene chloride and 100 μl of sodium borate buffer were added. After mixing and centrifuging at 2000 *g* for 2 min the organic layer was collected and evaporated to dryness at 40°C under a mild stream of nitrogen. The residue was redissolved in 100 μl of triethylamine solution (1 mmol $l^{-1}$ triethylamine in dichloroethane).

GC-MS analyses were performed using electron ionisation on a Varian 3400 gas chromatograph with a Finnigan MAT Incos 500 mass selective detector (Finnigan MAT, Veenendaal, The Netherlands). A fused silica permabond SE-52 GC-column, 25 m × 0.25 mm ID, film thickness 0.25 μm (Gimex, Geldermalsen, The Netherlands) was used. The column oven temperature was programmed from 80°C (after a 1 min hold) to 300°C rate of temperature increase 20°C $min^{-1}$, final hold 3 min. The injector and transferine temperatures were 240°C and 275°C respectively. The sample (1 μl) was injected using a Finnigan MAT A200S autosampler.

Two calibration curves (concentration range 0.5-250 ng $ml^{-1}$ and 250-2500 ng $ml^{-1}$) were constructed by plotting the peak height ratios (cotinine-cotinine-d3; ions 176:179) against the cotinine concentrations.

The limit of detection was 0.5 ng $ml^{-1}$. The between-days variability for the concentration range 0.5-30 ng $ml^{-1}$ was 20-40%, depending on the concentration ($n = 10$). The between-days variability at a level of 100 ng $ml^{-1}$ cotinine was 10% ($n = 10$).

The measurement of the creatinine concentrations was done automatically by the Jaffe alkaline picrate reaction, using the COBAS B10 centrifugation-analyser. (Roche Diagnostic Systems, Mijdrecht, The Netherlands). The within-day variation was 2% and the between-days variation was 3% for concentrations between 0.5 and 15 mmol $l^{-1}$.

### Data analysis

At the Department of Epidemiology, University of Utrecht, data analysis was performed with an Olivetti PCS 33 using SPSS version 4.0.1.

To explore the relationship between lung cancer risk and cotinine excretion values which reflects presumably the complete range of nicotine intakes, we divided the declared non-smokers as well as the smokers into tertiles of cotinine excretion. Odds ratios of strata for active smokers relating to lung cancer risk were calculated, defining the sum of the passive smokers and non-smokers as the reference group. To calculate the odds ratios of the strata relating to lung cancer risk for passive smokers, we defined the lowest tertile of the non-smokers as the reference group. The two highest tertiles of the non-smoking group were defined as passive smokers. Odds ratios were also calculated for different types of lung carcinoma.

In all analyses subjects with unreported cigarette consumption were excluded.

### Results

### Urinary excretion of cotinine according to declared smoking habits

The frequency distributions of urinary logarithmically transformed urinary cotinine concentrations of all women (controls plus cases) according to their declared smoking status at the time of urine collection are shown in Figure 1. Clearly there exists a high degree of association between their self-reported smoking status and the objective tests of cotinine excretion.

Comparing the distribution of urinary cotinine concentrations with declared smoking status (Figure 1) indicated that the optimal cut-off point for distinguishing between active smokers on the one hand and passive smokers and non-smokers on the other hand was at a level of 70 ng $ml^{-1}$ ($\log_{10}$ 70 being 1.85).

In order to allow for the influence of diuresis we have expressed the cotinine results not only as concentrations per ml of urine but also in the form of ratios on a creatinine basis. The correlation between both ways of expressing results is high ($r = 0.97$, 95% CI 0.96-0.97). The best cut-off point between smokers and non-smokers proved to be 100 ng $mg^{-1}$ of creatinine with a sensitivity of 91% and a specificity of 97% (questionnaire data acting as golden standard).

From a sample of 167 women (those representing the first wave of cases and controls; see Methods) cotinine has been measured in urine collected at two different points over time, with a 1 year interval. The Pearson correlation coefficient of the relation between the two samples concerning log cotinine (in ng $mg^{-1}$ creatinine) proved to be high ($r = 0.88$, 95% CI 0.83-0.91); above the level of 100 ng $mg^{-1}$ creatinine it was 0.79 (95% CI 0.62-0.89), below that level it was 0.49 (95% CI 0.32-0.64).

### Cotinine excretion and lung cancer risk

In investigating the relationship between the urinary excretion of cotinine and lung cancer risk a breakdown of exposures has been chosen so as to allow the study of lung cancer risk not only among active smokers but also among passive smokers. Thus three levels below 100 ng cotinine per mg creatinine and three levels above that point were distinguished. The results are shown in Tables I and II. The data show a clear trend of increasing lung cancer risk with rising cotinine excretion. The increased risk also appears to begin at levels of nicotine intake in the passive smoking range (Table II). It was found that adjusting for creatinine resulted in

**Figure 1** Frequency distribution of log cotinine concentration according to declared smoking status at time of urine collection (lung cancer cases included). ■, smokers; □, non-smokers.

**Table I** Risk of lung cancer according to urinary excretion of cotinine in active smokers

| Urinary cotinine in ng $mg^{-1}$ creatinine: range (geometric mean) | Lung cancer cases | Controls | Odds ratio (95% CI) |
|---|---|---|---|
| <100[a] (13) | 23 | 191 | 1.0 |
| 100–900 (372) | 5 | 32 | 1.3 (0.5–3.7) |
| 901–2251 (1429) | 21 | 17 | 10.3 (4.7–22.2) |
| 2260–7345 (3184) | 20 | 17 | 9.8 (4.5–21.3) |
| All smokers (1194) | 46 | 66 | 5.8 (3.3–10.3) |
| Total | 69 | 257 | |

[a]definition for passive smokers and non-smokers.

**Table II** Risk of lung cancer according to urinary excretion of cotinine in passive smokers

| Urinary cotinine in ng $mg^{-1}$ creatinine: range (geometric mean) | Lung cancer cases | Controls | Odds ratio (95% CI) |
|---|---|---|---|
| <9.2 (3.6) | 4 | 67 | 1.0 |
| 9.2–23.4 (14.9) | 10 | 62 | 2.7 (0.8–9.1) |
| 23.4–100 (41.8) | 9 | 62 | 2.4 (0.7–8.3) |
| Total | 23 | 191 | |

**Table III** Frequency distribution of lung carcinoma by histological subtype (cancers reported to the Cancer Registry)

| | Cases | |
|---|---|---|
| Histological subtype | n | % |
| Adenocarcinoma | 22 | 45 |
| Small-cell carcinoma | 13 | 27 |
| Squamous cell carcinoma | 8 | 16 |
| Carcinoid | 3 | 6 |
| Other | 3 | 6 |
| Total | 49 | 100 |

**Table IV** Odds ratios in relation to cotinine excretion for different types of lung cancer

| Cotinine (ng $mg^{-1}$ creatinine) | OR adenocarcinomas (95% CI) | OR other carcinomas (95% CI) |
|---|---|---|
| <100[a] | 1.0 | 1.0 |
| 100–900 | 0.3 (0.0–2.2) | 4.7 (1.1–20.1) |
| 901–2251 | 12.4 (2.3–67.1) | 9.3 (2.5–34.8) |
| 2260–7345 | 3.1 (0.4–24.6) | 15.2 (4.2–54.7) |
| All smokers | 2.1 (0.8–5.9) | 9.3 (3.4–25.9) |

[a]definition for passive smokers and non-smokers. OR, odds ratios.

somewhat higher odds ratios concerning lung cancer risk than without such adjustment (data not shown).

The lung cancer distribution between the different levels of self-reported cigarette consumption did not differ significantly ($P = 0.14$; $\chi^2$ test) from the one of the corresponding cotinine categories. Urinary cotinine did not seem to be more predictive of lung cancer risk than self-reported cigarette consumption.

*Histological type and lung cancer risk in relation to smoking*

Data on histological type of lung cancer were available through the cancer registry for 49 of the patients (see Table III). Relative risk in relation to cotinine excretion was computed separately for adenocarcinoma in contrast to the sum of other histological types.

The results presented in Table IV show that the relationship with smoking is much weaker for adenocarcinoma of the lung than for the other pulmonary cancers. In fact, an odds ratio significantly above 1 was found only at fairly high levels of nicotine intake.

### Discussion

This is believed to be among the first studies of its kind to establish a relationship between exposure to tobacco smoke and lung cancer risk by direct and objective methods, i.e. measuring cotinine in urinary samples from a cohort of women followed for a period of up to 15 years. The results confirm recent insight that lung cancer risk ratios according to amount smoked are equally high in women as in men (Garfinkel and Stellman, 1988; Osann *et al.*, 1993). Unfortunately, no personal data were available on the duration of smoking by the women of the cohort. However, we have reason to believe that they are from a generation which began smoking immediately after World War II (when they were 20–35 years of age), and the high relative risks of lung cancer experienced by the smokers during the years 1977–91 seem to substantiate this belief.

A salient aspect of the present paper is concerned with the possible risk of environmental tobacco smoke. The results of this study seem to indicate that there is not only a very high lung cancer risk associated with active smoking as expressed by high levels of cotinine excretion but also a modest increase of risk at levels of cotinine associated with passive smoking. The relative risk in passive smokers according to our classification (having a reference group with a minimal cotinine excretion) was found to be of the order of 2 to 3 rather that 1.3 or 1.5 (the meta-estimate by Wald *et al.*, 1986) but the confidence intervals are wide (Table II). It is more in line with the relatively high estimates published by Garfinkel *et al.* (1985), Pershagen *et al.* (1987) and some of the papers mentioned by Blot and Fraumeni (1986).

With our GC–MS method, cotinine excretion measurements were slightly higher than those reported by some others (Jarvis *et al.*, 1984; Wald *et al.*, 1984; Wall *et al.*, 1988; Haley *et al.*, 1989). However, they were much lower than those reported by Matsukura *et al.* (1984). It should be borne in mind that excretion levels corrected for creatinine excretion are affected by gender since women have less muscle mass then men.

The question could be raised whether cotinine levels reflect passive smoking (Idle, 1984). However, Jarvis *et al.* (1984), Wald *et al.* (1984), Haley *et al.* (1989), Riboli *et al.* (1990) and Cook *et al.* (1994) have shown that in non-smokers elevated cotinine levels are directly related to an increased environmental exposure to tobacco smoke. In a recent study Hecht *et al.* (1993) found a high correlation between urinary cotinine and a powerful tobacco-specific lung carcinogen. There is no reason to believe that the women were lying about their smoking habits when they were visiting the screening facility in good health. Biochemical errors are unlikely since the method of cotinine determination is very specific. We rule out any genetic traits to produce cotinine naturally. Theoretically, cotinine excretion due to the consumption of vegetables could complicate the interpretation of our study. However, cotinine exposure from vegetables is greatly reduced when vegetable skins, which contain most of the nicotine, are not eaten or cooked in water, thereby extracting the nicotine. Moreover, absorption of nicotine from the stomach is poor and 70% of it is metabolised during its first pass through the liver (Henningfield, 1993). Thus the most likely explanation of low levels of urinary cotinine is exposure to environmental tobacco smoke.

Since cotinine excretion levels obtained at intake in the cohort correlated well with those obtained 1 year later, there is objective evidence for the notion that these levels reflect the chronic smoking habits of the persons concerned and the relative constancy of levels of tobacco smoke in their immediate human environment. Jarvis *et al.* (1987) came to a similar conclusion after studying cotinine in saliva. It was shown by an independent laboratory that cotinine in the urinary samples of our 'bank' (which were kept frozen at −20°C) did not deteriorate over a period of 9 years (Riboli *et al.*, 1995).

In our study, urinary cotinine did not seem to be more predictive of lung cancer, than self-reported cigarette consumption. This was not surprising since the urine samples and data about reported cigarette comsumption were collected before the risk of smoking in women became an important issue, and therefore probably very reliable. Unfortunately, no information was solicited concerning self-reported passive smoke exposure since data from the questionnaires were collected long before the first papers on that issue were published. So, it was not possible to study whether cotinine is more predictive of lung cancer risk than self-reported passive smoke exposure. However, there is an advantage of having an objective test of exposure to tobacco smoke instead of a questionnaire about hours of passive smoke exposure in non-smokers.

The present paper confirms results published by others (Kreyberg, 1954; Trichopoulos *et al.*, 1981; Damber and Larson, 1986; Koo *et al.*, 1987; Pershagen *et al.*, 1987; Osann, 1991; Brownson *et al.*, 1992) on the relationship between smoking and adenocarcinoma of the lung; it is much weaker than that of other histological types. In fact, no relationship between adenocarcinoma and passive smoking was apparent from the data. The relatively high proportion of adenocarcinoma found in this cohort is in agreement with the relatively low percentage of smokers (30); the smoking-related epidemic of lung cancer in women probably is only at its beginning.

In this study a direct and objective method is used to explore the relationship between exposure to tobacco smoke and lung cancer risk. There is some advantage in an approach which does not require either histories of active smoking or histories concerning possible exposure to environmental tobacco smoke.

**Acknowledgements**
The authors are indebted to J Fracheboud, FJJ Bosman and CHF Gimbrère of the Comprehensive Cancer Centre (IKMN) at Utrecht for providing data on lung cancer mortality and incidence respectively, to RK Vermeulen of the National Institute of Public Health and Environmental Protection for expert and analytical assistance and to G de Groot for his contribution to the cotinine determinations in the early phase of the study. The project was subsidised partly by the Prevention Fund (no. 28-2154), the Hague and by the Dutch Chief Medical Inspectorate of Health.

**References**

BLOT WJ AND FRAUMENI JF. (1986). Passive smoking and lung cancer. *J. Natl Cancer Inst.*, **77,** 993–1000.
BROWNSON RC, CHANG JC AND DAVIS JR. (1992). Gender and histologic type variations in smoking-related risk of lung cancer. *Epidemiology*, **3,** 61–64.
COOK DG, WHINCUP PH, JARVIS MJ, STRACHAN DP, PAPACOSTA O AND BRYANT A. (1994). Passive exposure to tobacco smoke in children aged 5–7 years: individual, family and community factors. *Br. Med J.*, **306,** 384–388.
DAMBER LA AND LARSON LG. (1986). Smoking and lung cancer, with special regard to type of smoking and type of cancer. A case–control study in N Swden. *Br. J. Cancer*, **53,** 573–681.
DE WAARD F, ROMBACH JJ, COLLETTE HJA AND HONING C. (1984). The DOM project for the early detection of breast cancer, Utrecht, the Netherlands. *J. Chron. Dis.*, **37,** 1–44.
DEN TONKELAAR I, BLANKENSTEIN MA, COLLETTE HJA, DE WAARD F AND THYSSEN JHH. (1989). A prospective study on corpus luteum function and breast cancer risk. *Gynecol. Endocrinol.*, **3,** 11–19.
DOLL R AND PETO R. (1978). Cigarette smoking and bronchial carcinoma: dose and time relationships among regular smokers and life-long non-smokers. *J. Epidemiol. Community Health*, **32,** 303–313.
DOLL R, GRAY R, HAFNER B AND PETO R. (1980). Mortality in relation to smoking: 22 years' observations in female British doctors. *Br. Med. J.*, **280,** 967–971.
GARFINKEL L AND STELLMAN SD (1988). Smoking and lung cancer in women: findings in a prospective study. *Cancer Res.*, **48,** 6951–6955.
GARFINKEL L, AUERBACH O AND JOUBERT L. (1985). Involuntary smoking and lung cancer: a case–control study. *J. Natl Cancer Inst.*, **75,** 463–469.
HALEY NJ, COLOSIMO SG, AXELRAD CM, HARRIS R AND SEPKOVIC DW. (1989). Biochemical validation of self-reported exposure to environmental tobacco smoke. *Environ. Res.*, **49,** 127–135.
HECHT SS, CARMELLA SG, MURPHY SE, AKERKAR S, BRUNNEMANN KD AND HOFFMANN D. (1993). A tobacco-specific lung carcinogen in the urine of men exposed to cigarette smoke. *N. Engl. J. Med.*, **329,** 1543–1546.
HENNINGFIELD JE. (1993). More on the nicotine content of vegetables (letter). *New Engl. J. Med.*, **329,** 1581.
IDLE JR. (1984). Titrating exposure to tobacco smoke using cotinine – a minefield of misunderstandings. *J. Clin. Epdiemiol.*, **43,** 313–317.
JARVIS MJ. (1989). Application of biochemical intake markers to passive smoking: measurement and risk estimation. *Mutat. Res.*, **222,** 101–110.
JARVIS MJ, TUNSTALL-PEDOE H, FEYERABEND C, VESEY C AND SALLOOJEE Y. (1984). Biochemical markers of smoke absorption and self-reported exposure to passive smoking. *J. Epidemiol. Community Health*, **38,** 335–339.
JARVIS MJ, McNEILL AD, RUSSELL MAH, WEST RJ, BRYANT A AND FEYERABEND C. (1987). Passive smoking in adolescents: one-year stability of exposure in the home. *Lancet*, **1,** 1324–1325.
KOO LC, HO JHC, SAW D AND HO C. (1987). Measurements of passive smoking and estimates of lung cancer risk among non-smoking Chinese females. *Int. J. Cancer*, **39,** 162–169.
KREYBERG L. (1954). Significance of histological typing in study of epidemiology of primary epithelial lung tumours: study of 466 cases. *Br. J. Cancer*, **8,** 199–208.
MATSUKURA S, TAMINATO T, KITANO N, SEINO Y, HAMADA H, UCHIHASHI M, NAKAJIMA H AND HIRATA Y. (1984). Effects of environmental tobacco smoke on urinary cotinine excretion in nonsmokers. *N. Engl J. Med.*, **311,** 828–832.
OSANN KE. (1991). Lung cancer in women: the importance of smoking, family history of cancer and medical history of respiratory disease. *Cancer Res.*, **51,** 4893–4897.
OSANN KE, ANTON-CULVER H, KUROSAKI T AND TAYLOR T. (1993). Sex differences in lung cancer risk associated with cigarette smoking. *Int. J. Cancer*, **54,** 44–48.
PARKIN DM. (1989). Trends in lung cancer incidence worldwide. *Chest*, **96,** 5S–8S.
PERSHAGEN G, HRUBEC Z AND SVENSSON C. (1987). Passive smoking and lung cancer in Swedish women. *Am. J. Epidermol.*, **125,** 17–24.
POORTMAN J, VAN DER SMISSEN J, COLLETTE HJA AND DE WAARD F. (1979). Ratio of 11-desoxy 17-oxosteroids to creatinine in a population screened for breast cancer. *Br. J. Cancer*, **39,** 688–695.
RIBOLI E, PRESTON-MARTIN S AND SARACCI R. (1990). Exposure of non-smoking women to environmental tobacco smoke: a 10-country collaborative study. *Cancer Causes Control*, **1,** 243–252.
RIBOLI E, HALEY NJ, DE WAARD F AND SARACCI R. (1995). Validity of urinary biomarkers of exposure to tobacco smoke following prolonged storage. *Int. J. Epidemiol.*, **24,** 354–358.
SHERMAN BM AND KORENMAN SG. (1974). Inadequate corpus luteum function: a pathophysiological interpretation of human breast cancer epidemiology. *Cancer*, **33,** 1306–1312.
TRICHOPOULOS D, KALANDIDI A AND SPARROS L. (1981). Lung cancer and passive smoking: conclusions of Greek study. *Int. J. Cancer*, **27,** 1–4.
TWEEDIE RL AND MENGERSEN KL. (1992). Lung cancer and passive smoking: reconciling the biochemical and epidemiological approaches. *Br. J. Cancer*, **66,** 700–705.
WALD NJ, BORCHAM J, BAILEY A, RITCHIE C, HADDOW JE AND KNIGHT G. (1984). Urinary cotinine as marker of breathing other peoples' tobacco smoke. *Lancet*, **1,** 230–231.
WALD NJ, NANCHAHAL K, THOMSON SG AND CUCKLE HS. (1986). Does breathing other peoples' tobacco smoke cause lung cancer? *Br. Med J.*, **293,** 1217–1222.
WALL MA, JOHNSON J, JACOB P AND BENOWITZ NL. (1988). Cotinine in the serum, saliva and urine of nonsmokers, passive smokers and active smokers. *Am. J. Public Health*, **78,** 699–701.